# *ALFI*: Cell cycle phenotype annotations of label-free time-lapse imaging data from cultured human cells

**DOI:** 10.1038/s41597-023-02540-1

**Published:** 2023-10-04

**Authors:** Laura Antonelli, Federica Polverino, Alexandra Albu, Aroj Hada, Italia A. Asteriti, Francesca Degrassi, Giulia Guarguaglini, Lucia Maddalena, Mario R. Guarracino

**Affiliations:** 1 ICAR, Institute for High-Performance Computing and Networking, National Research Council, Naples, Italy; 2 IBPM, Institute of Molecular Biology and Pathology, National Research Council, Rome, Italy; 3 Department of Economics and Law, University of Cassino and Southern Lazio, Cassino, Italy; 4 Laboratory of Algorithms and Technologies for Networks Analysis, National Research University Higher School of Economics, Moscow, Russia

**Keywords:** Mitosis, Cellular imaging

## Abstract

Detecting and tracking multiple moving objects in a video is a challenging task. For living cells, the task becomes even more arduous as cells change their morphology over time, can partially overlap, and mitosis leads to new cells. Differently from fluorescence microscopy, label-free techniques can be easily applied to almost all cell lines, reducing sample preparation complexity and phototoxicity. In this study, we present *ALFI*, a dataset of images and annotations for label-free microscopy, made publicly available to the scientific community, that notably extends the current panorama of expertly labeled data for detection and tracking of cultured living nontransformed and cancer human cells. It consists of 29 time-lapse image sequences from HeLa, U2OS, and hTERT RPE-1 cells under different experimental conditions, acquired by differential interference contrast microscopy, for a total of 237.9 hours. It contains various annotations (pixel-wise segmentation masks, object-wise bounding boxes, tracking information). The dataset is useful for testing and comparing methods for identifying interphase and mitotic events and reconstructing their lineage, and for discriminating different cellular phenotypes.

## Background & Summary

Advances in computer in vision, imaging technologies, and computational tools have led to significant results in video event detection and recognition. Many algorithms have been developed to tackle events arising in different video domains, such as abnormality detection for surveillance^1^, human action detection^2^, and cell population monitoring^3^ in several biological and biomedical studies. In cell biology studies, time-lapse microscopy advanced methodologies are used to observe the dynamic behavior of single cells over time^4^. The analysis of image sequences enables the quantification of relevant parameters, such as the number of cells and their geometric features (e.g., perimeter, area, nuclear position and shape roundness), as well as the assessment of their movement and fate, gaining unique spatio-temporal information on dynamic biological processes^5^. The drug discovery and cancer research fields have largely benefited from these innovative approaches^6,7^. Among anti-cancer compounds, an important class is represented by anti-mitotic drugs, which arrest cell division (mitosis) and induce cell death^8^. Indeed, monitoring by time-lapse microscopy the single-cell response to anti-mitotic drugs in terms of cell division and cell death has allowed revealing the heterogeneity of induced events within a cell population, thus providing key information for the characterization of novel compounds of therapeutic interest^9^. In addition, abnormal mitotic progression detected by time-lapse microscopy may help identify mitotic regulators and/or pathways impinging on mitosis, which can widen our understanding of the genetic regulation of cell proliferation and death and can represent novel targets for anti-cancer drugs. The application of such analyses to extended datasets, such as those obtained in high-throughput experiments, requires automated approaches based on machine learning/artificial intelligence with the aim of defining complex phenotypic profiles^10,11^. Available automated systems for detecting cellular events such as cell division and cell death mostly rely on the use of fluorescently labeled cells^12,13^ limiting the flexibility of applications due to phototoxicity effects, cell lines availability, and fluorescent probe stability. One way to minimize these issues is to work with label-free imaging techniques^14^, such as phase-contrast and differential interference contrast (DIC), enabling long-term monitoring of live and intact cells by time-lapse experiments. In particular, the DIC technique produces a pseudo-3D effect and shadow-cast images, and is well suited for the reliable identification of cell division and cell death events, and specific mitotic phenotypes. Thus, automated systems for analyzing these cellular events under label-free microscopy and datasets of cell annotations to compare results and improve the overall procedures are of great interest.

A recent survey paper^14^ provides an updated summary of available annotated data for various tasks involving label-free microscopy images, including the Cell Tracking Challenge^15^, EVICAN^16^, LIVECell^17^, and the Phase Contrast Time-lapse Microscopy Datasets^18^. In this increasing amount of valuable annotated data, we noticed that DIC-acquired images, nontransformed cell lines and experimental conditions to increase the numerosity of useful examples for cell proliferation/cell death analyses, are still poorly represented. Here, we present *ALFI* (Annotations for Label-Free Images), a dataset of sequences and annotations for DIC microscopy imaging, made publicly available to the scientific community through figshare^19^. In this dataset, we collected image sequences from live samples from both nontransformed and cancer human cell lines. Experimental conditions include asynchronously growing cells, cell cycle synchronization methods, and treatments with anti-mitotic drugs. Furthermore, we focused on segmentation, tracking, and lineage of dividing cells and classification of cell division/cell death-related phenotypes. Given the relevance of detecting cellular effects of newly identified anti-mitotic compounds in pre-clinical cancer studies, increasing the amount of expertly labeled datasets may significantly contribute to the successful development of dedicated cell segmentation, detection, classification, and tracking algorithms by supervised learning.

The *ALFI* image dataset consists of 29 original videos and sequences (2366 DIC images) for a total of 14272 minutes from time-lapse experiments, deriving from the recording of different human nontransformed and cancer cell lines untreated or exposed to known anti-mitotic drugs. Various types of annotations are provided: (i) pixel-wise segmentation masks (796 frames, 16564 cells) for cell segmentation and for discriminating interphase and mitotic cells; (ii) object-wise bounding box annotations for the classification of interphase and mitotic cells (796 frames, 16564 cells) and for the classification of non-interphase cells into four different phenotypes (1926 frames, 7518 cells); and (iii) annotations for tracking interphase and mitotic cells (331 tracks, 16564 cells) and non-interphase cells of the four phenotypes (357 tracks, 7518 cells). Given these numbers, the *ALFI* dataset notably extends the panorama of existing annotated datasets^14,18^ for DIC time-lapse microscopy, besides providing unprecedented annotated data for classifying cells displaying division-related phenotypes.

## Methods

### *ALFI* image dataset

**Fig. 1:**
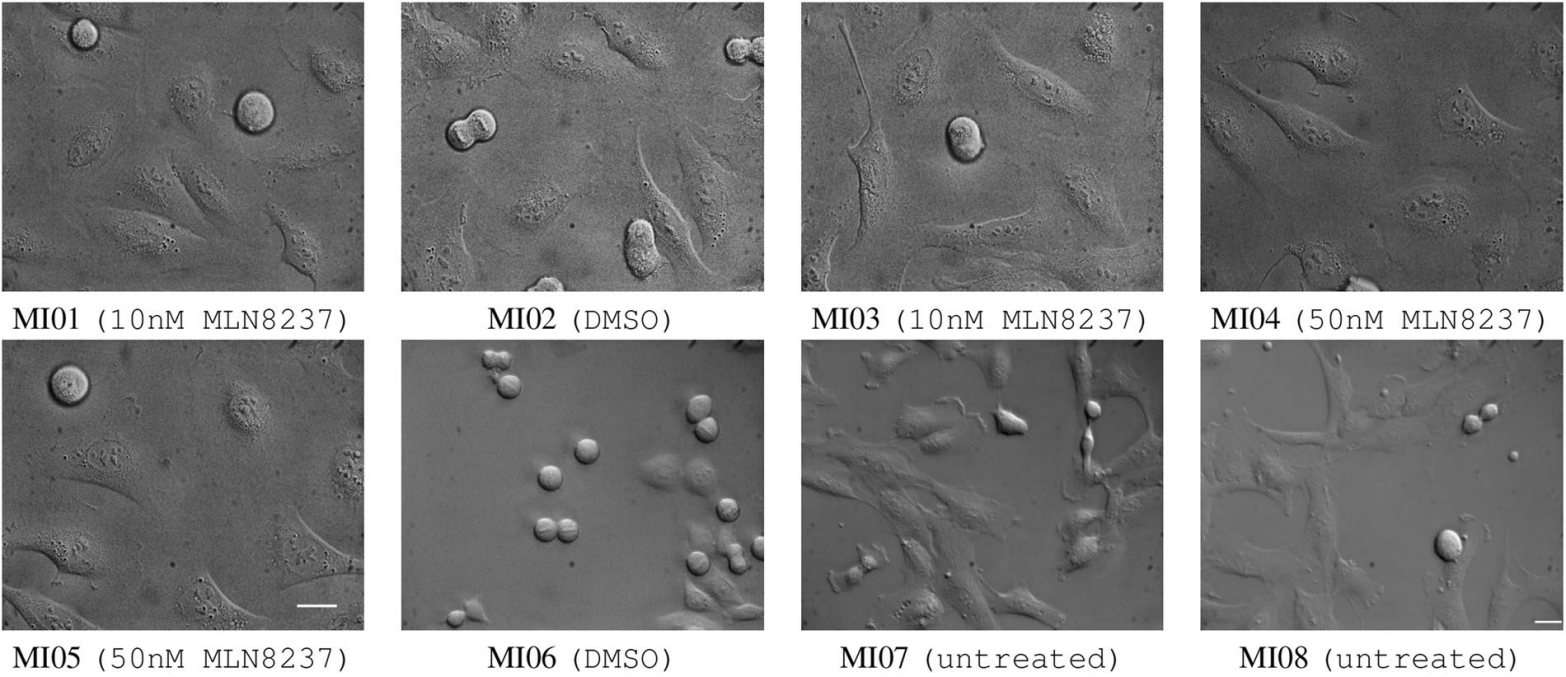
The *ALFI* dataset: the first frame of sequences MI01-MI08 used in both *Task 1* and *Task 2* is shown. U2OS (MI01-MI05) or HeLa (MI06) cultures were treated with 0.1% DMSO or MLN8237 at different concentrations, as specified in the captions. MI07 and MI08 represent hTERT RPE-1 cells in untreated conditions. All the frames were equalized for better visualization. The scale bar for MI01-MI05 is shown in MI05 and that for MI06-MI08 is shown in MI08. Both scale bars represent 25 *μ*m.

**Fig. 2:**
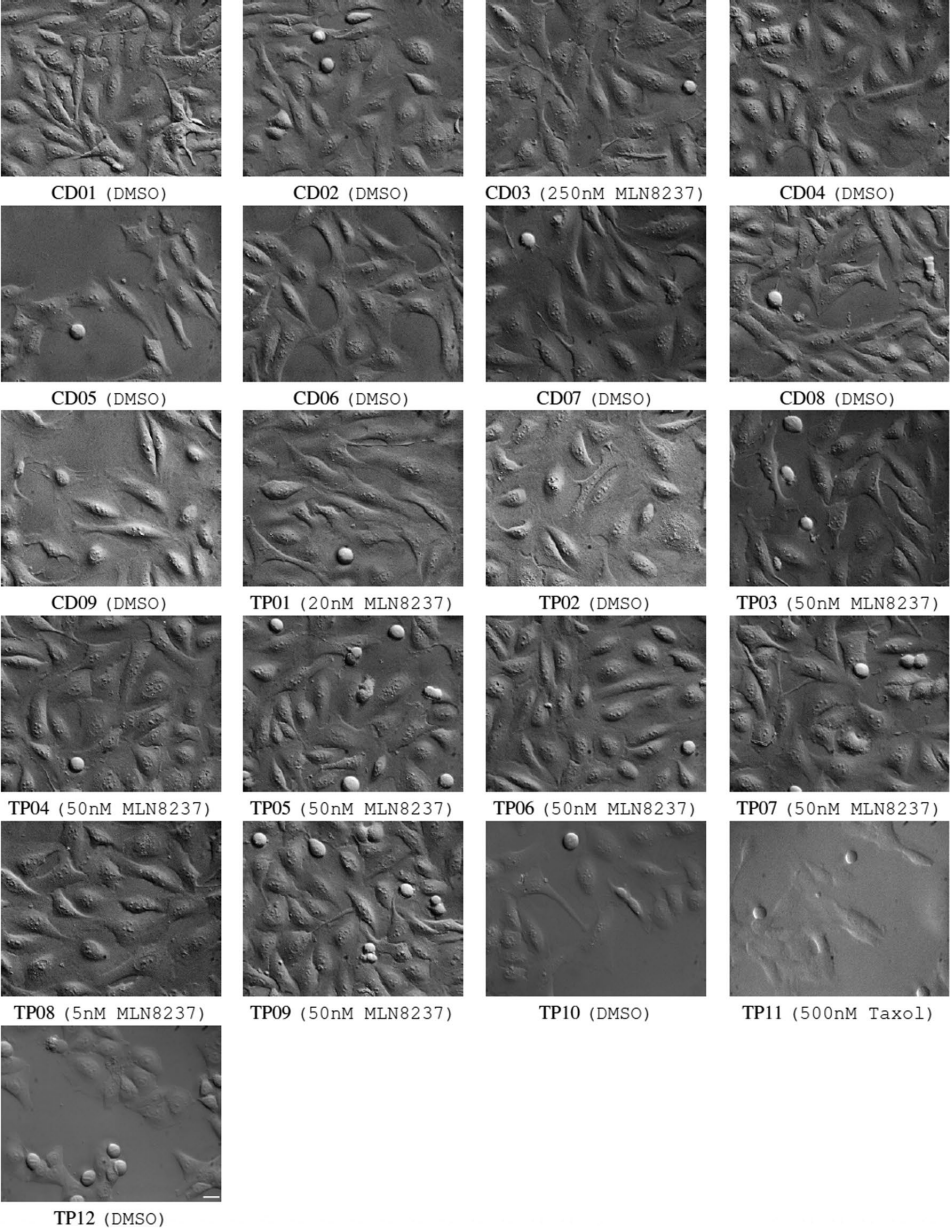
The *ALFI* dataset: the first frame of sequences used for *Task 2* is shown. U2OS (CD01-CD09 and TP01-TP10) or HeLa (TP12) cultures were treated with DMSO 0.1% or MLN8237 at different concentrations, as specified in the captions. TP11 represents hTERT RPE-1 cells treated with 500 nM Taxol. All the frames were equalized for better visualization. Scale bar: 25 *μ*m.

The *ALFI* image dataset is made available as an open-source dataset, which contains 29 original time-lapse microscopy videos and image sequences, named MI01-MI08, CD01-CD09, and TP01-TP12, of the following human cell lines: retinal pigment epithelial nontransformed immortalized hTERT RPE-1, uterine cervical adenocarcinoma HeLa, osteosarcoma U2OS (ATCC: HTB-96) cells and U2OS cells stably expressing histone H2B-GFP and RFP-alpha-tubulin (indicated as “U2OS fluo” in Tables 1–3). Cells were grown at 37 °C and 5% CO_2_ in complete DMEM (HeLa, U2OS) or DMEM/F12 (hTERT RPE-1) medium, supplemented with 10% fetal bovine serum. Cells were passaged every two-three days and kept in culture for no more than four weeks. To enrich in mitotic figures, cell cycle synchronization protocols were used. Briefly, HeLa and U2OS cells were synchronized by a 24-hour block in 2 mM thymidine. Cultures were then released from the G1/S arrest by washout of thymidine and replacement with fresh medium containing 30 μM deoxycytidine; 6 hours post-release cultures were video recorded immediately after treating them with solvent only (dimethyl sulfoxide, DMSO) or Aurora kinase inhibitor (Alisertib - MLN8237, Selleck Chemicals) at different concentrations, as specified in Figs. 1, 2, to induce mitotic abnormalities and cell death. Asynchronously growing hTERT RPE-1 were either untreated or treated with DMSO or 500 nM Taxol (Sigma Aldrich) (see Figs. 1, 2). Cells seeded in 2-4-8 wells micro-slides (Ibidi, 80286, 80426, 80826) were recorded every 5 minutes (CD01-CD09 and TP01-TP10) or every 7 minutes (MI01-MI08, TP11, and TP12), under an inverted microscope (Eclipse Ti, Nikon) equipped with a DS-Qi1Mc camera (Nikon) and stage incubator (basic WJ, Okolab) to keep constant environmental conditions during the whole registration. 40× (S Plan Fluor, N.A. 0.60, DIC) or 60× Oil (Plan Apo, N.A.1.4, DIC) Nikon objectives were used, as specified in Table 1. The videos have been generated over the years in our laboratories, with experimental conditions set up for investigating mitotic regulators and anti-mitotic compounds, and have proven highly informative in past studies^6,20,21^. Example frames are shown in Figs. 1, 2 and details are reported in Tables 2, 3.

**Table 1:** Description of cell lines and microscope parameters used for each recorded sequence.

Sequence(s)	Cell line	Objective lens	pixel size
MI01-MI05	U2OS fluo	60×	0.18 *μm*/*px*
CD01-CD09 and TP01-TP10	U2OS	40×	0.26 *μm*/*px*
MI06 and TP12	HeLa	40×	0.26 *μm*/*px*
MI07, MI08, and TP11	hTERT RPE-1	40×	0.26 *μm*/*px*

**Table 2:** Details on sequences MI01-MI08 and their annotations for *Task 1* (discriminating interphase and mitotic cells, and their progeny).

Seq. name	Cell line	Duration (mins)	# frames	# annotated frames	# mitotic cells	# interphase cells	Total # cells	Total # tracks
MI01	U2OS fluo	483	69	69	99	837	936	19
MI02	U2OS fluo	483	69	69	40	913	953	21
MI03	U2OS fluo	483	69	69	118	765	883	21
MI04	U2OS fluo	483	69	69	203	353	556	10
MI05	U2OS fluo	483	69	69	176	564	740	18
MI06	HeLa	1393	199	199	740	5628	6368	89
MI07	hTERT RPE-1	1148	164	164	124	3892	4016	96
MI08	hTERT RPE-1	616	88	88	76	2036	2112	57
Tot.		5572	796	796	1576	14988	16564	331

**Table 3:** Details on all sequences and their annotations for *Task 2* (discriminating non-interphase cells into four phenotype classes).

Seq. name	Cell line	Duration (mins)	# frames	# annotated frames	# early mitoses	# late mitoses	# dead cells	# multi- polar cells	Total # cells	Total # tracks
CD01	U2OS	340	68	64	32	12	81	0	125	5
CD02	U2OS	375	75	74	116	48	32	0	196	8
CD03	U2OS	265	53	53	53	0	30	0	83	2
CD04	U2OS	255	51	47	0	0	94	0	94	2
CD05	U2OS	345	69	64	1	3	60	0	64	2
CD06	U2OS	330	66	57	18	12	49	0	79	5
CD07	U2OS	325	65	57	86	37	36	0	159	8
CD08	U2OS	280	56	56	119	21	102	0	242	6
CD09	U2OS	500	100	79	60	58	37	0	155	13
TP01	U2OS	250	50	50	131	2	0	19	152	8
TP02	U2OS	520	104	86	145	58	0	10	213	12
TP03	U2OS	265	53	53	197	43	0	8	248	12
TP04	U2OS	275	55	55	174	42	0	9	225	11
TP05	U2OS	315	63	63	139	42	2	4	187	11
TP06	U2OS	80	16	16	11	1	0	4	16	1
TP07	U2OS	265	53	53	91	22	0	7	120	6
TP08	U2OS	245	49	45	85	13	0	8	106	4
TP09	U2OS	330	66	66	302	46	0	25	373	17
TP10	U2OS	165	33	24	13	9	0	7	29	3
TP11	hTERT RPE-1	1771	253	253	2519	10	120	58	2707	36
TP12	HeLa	1204	172	131	488	30	87	5	610	92
MI01	U2OS fluo	483	69	44	62	20	0	0	82	5
MI02	U2OS fluo	483	69	21	16	14	0	0	30	5
MI03	U2OS fluo	483	69	66	68	30	0	0	98	6
MI04	U2OS fluo	483	69	61	184	11	0	0	195	5
MI05	U2OS fluo	483	69	69	130	24	0	0	154	5
MI06	HeLa	1393	199	111	465	164	0	0	629	41
MI07	hTERT RPE-1	1148	164	66	45	45	0	0	90	16
MI08	hTERT RPE-1	616	88	42	34	23	0	0	57	10
Tot.		14272	2366	1926	5784	840	730	164	7518	357

**Fig. 3:**
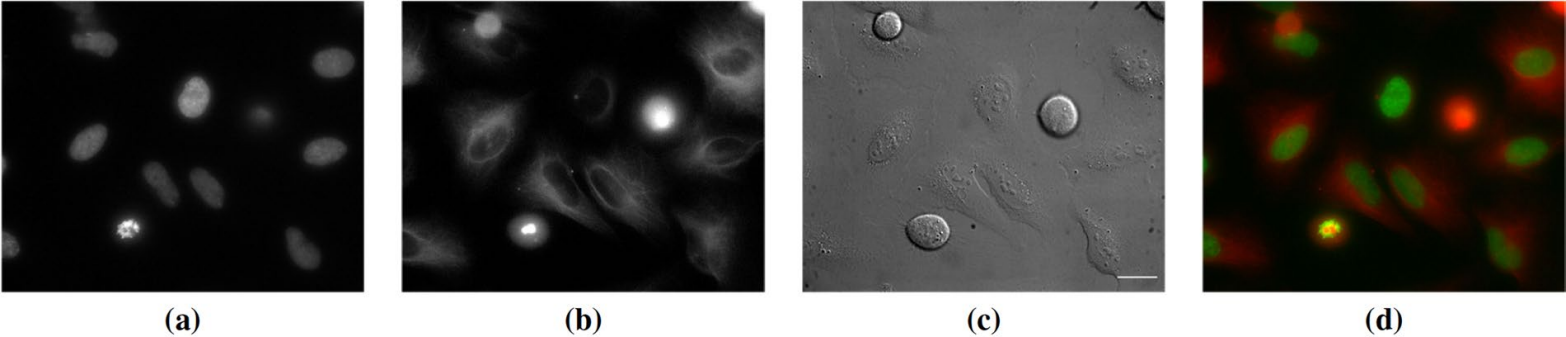
Example of fluorescence images from the MI01 sequence. (**a**) H2B-GFP, (**b**) RFP-alpha-tubulin, (**c**) DIC, and (**d**) merged fluorescence signals. For better visualization, contrast enhancement has been applied to (**a**–**c**). Scale bar: 25 *μ*m.

MI01-MI05 sequences also contain the fluorescence images of histone H2B-GFP and RFP-alpha-tubulin, as exemplified in Fig. 3. However, being interested in label-free non-phototoxic imaging, we considered only the DIC channel for extracting the image sequences; nonetheless, the interested user can extract the fluorescence images by the video files made available in the dataset.

The videos, stored in ND2 format, have 12-bit depth and 1280 × 1024 pixel resolution. For each video, the DIC channel was automatically equalized and its frames extracted into TIFF images using the NIS-Elements Viewer software. The frames were transformed into PNG images using the Fiji^22^ distribution of ImageJ^23^. Despite the equalization step, a few frames within the sequences may display brightness variations, due to environmental undesired sporadic changes that may occur during experiments. We performed annotations on these frames too, which may help to train algorithms to manage potential illumination variations.

### *ALFI* annotations

Annotations for the *ALFI* dataset have been produced for two different tasks: 1) discriminating and tracking interphases, mitoses, and their progeny, and 2) discriminating non-interphase cells into four phenotype classes.

#### *Task 1*: Discriminating interphases and mitotic cells, and their progeny

**Fig. 4:**
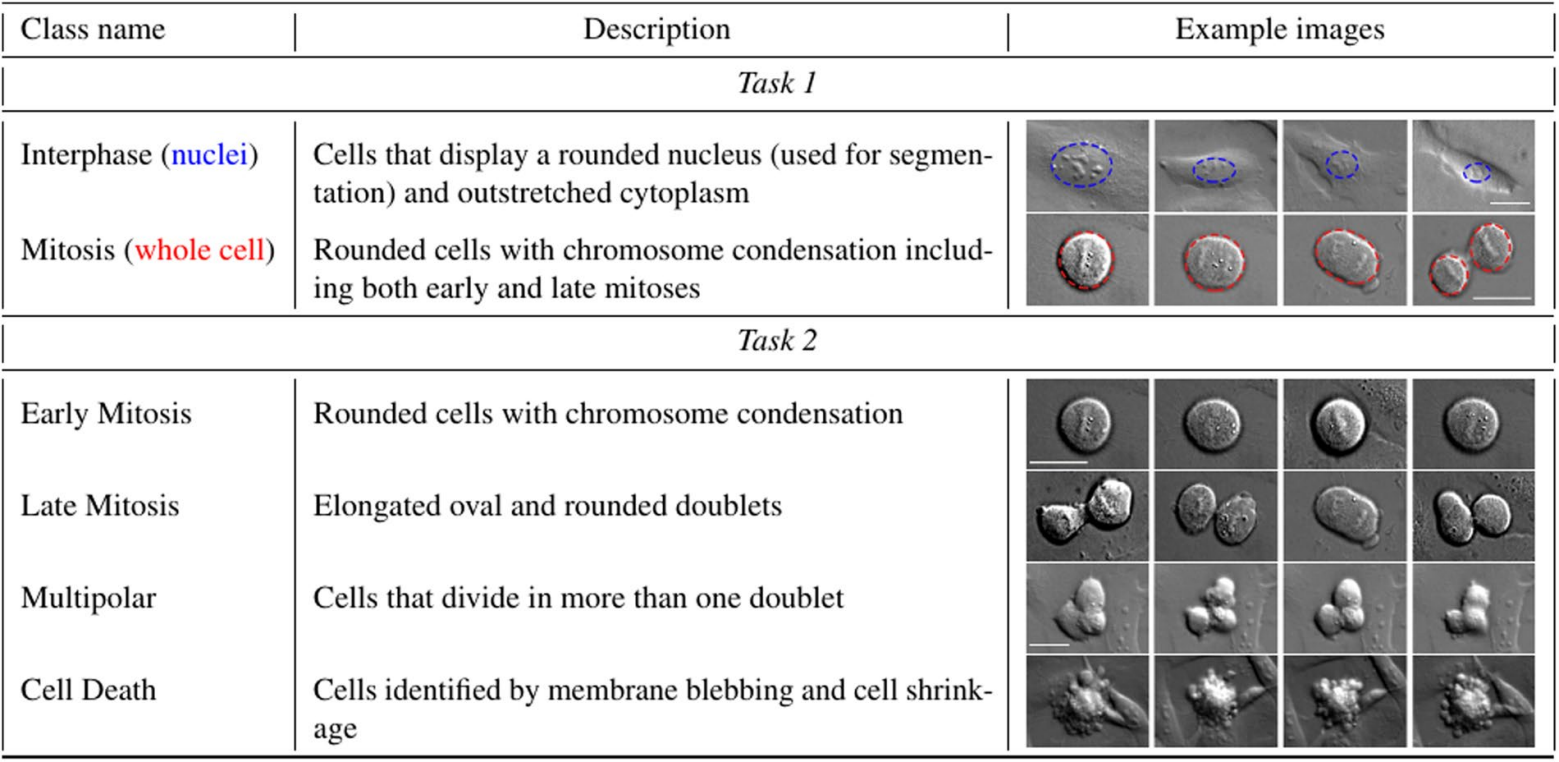
Description of the classes for *Task 1* (discriminating interphase and mitotic cells, and their progeny) and *Task 2* (discriminating non-interphase cells into four phenotype classes). The blue and red dotted lines in the example images for *Task 1* represent the nuclei (for interphase cells) and the whole cell area (for mitoses) used for segmentation, respectively. One scale bar applies to Early mitosis and Late mitosis. One scale bar applies to Multipolar and Cell Death. Scale bars: 25 *μ*m.

Under DIC time-lapse microscopy, interphase cells display a rounded nucleus and outstretched cytoplasm, while mitotic cells are recognized by cell rounding and chromosome condensation. If mitosis is successful, two daughter cells will emerge, and they will have the appearance of interphase cells. In this task, we discriminate between mitotic cells (that include both early and late mitosis) and interphase cells. A concise description and visualization of the cell categories analyzed in this task is given in the upper part of Fig. 4.

**Fig. 5:**
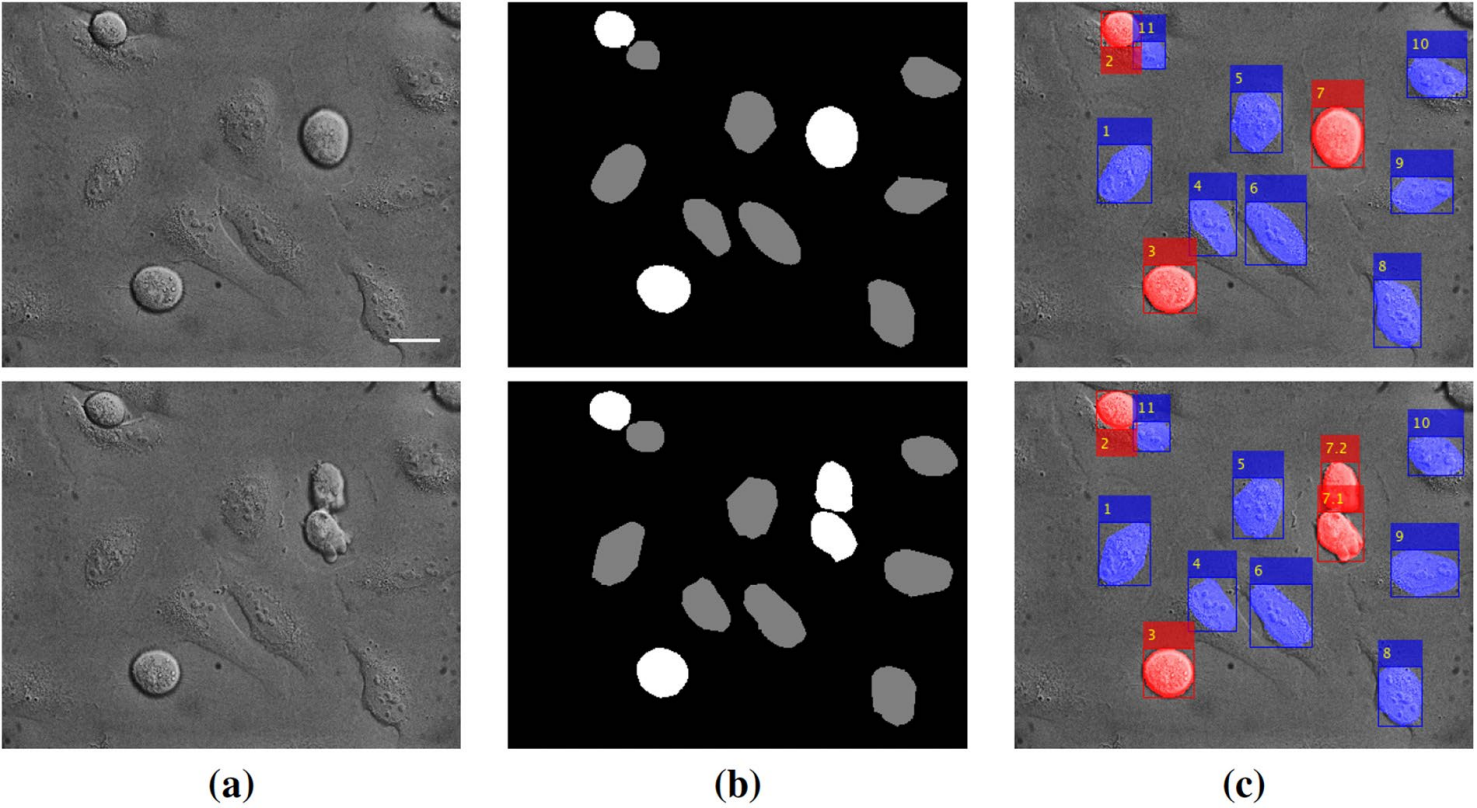
Examples of *ALFI* dataset annotations for *Task 1*. Sequence MI01, frames no. 4 (first row) and 5 (second row): (**a**) original images; (**b**) segmentation masks (black = *background*; grey = *interphase cells*; white = *mitotic cells*); (**c**) bounding boxes and tracking IDs for each interphase (blue) or mitotic (red) cell. Scale bar: 25 *μ*m.

Three types of annotations have been produced for sequences MI01-MI08: pixel-wise segmentation masks, object-wise bounding boxes, and object-wise tracking and lineage information. Examples of these annotations superimposed over the corresponding images are given in Fig. 5 for two frames of the MI01 sequence.

The segmentation masks render pixel-wise labeling of interphases (nuclei) and mitoses (whole cells) for each image. Examples are given in Fig. 5b, where mitotic cells are shown in white and interphase nuclei in grey, and in Fig. 4 with blue (nuclei) and red (whole cell) dotted lines. For each frame, the segmentation masks of interphase nuclei and mitoses have been obtained by producing an initial segmentation, using Matlab or ilastik^24^ tools, and then manually refined. The resulting masks have been carefully checked by four expert biologists (see Technical Validation).

The bounding boxes (position and size of the smallest box containing an interphase nucleus or a whole mitotic cell) have been extracted from the segmentation masks using Matlab. Examples are given in Fig. 5c, where interphase cells are delimited with blue bounding boxes and mitotic cells with red bounding boxes.

Tracking has been obtained by associating the bounding boxes of the same object in adjacent frames based on motion, estimated by a Kalman filter, using Matlab. This association is stored using an ID assigned to each cell consistently throughout the entire sequence (see Fig. 5c).

**Fig. 6:**
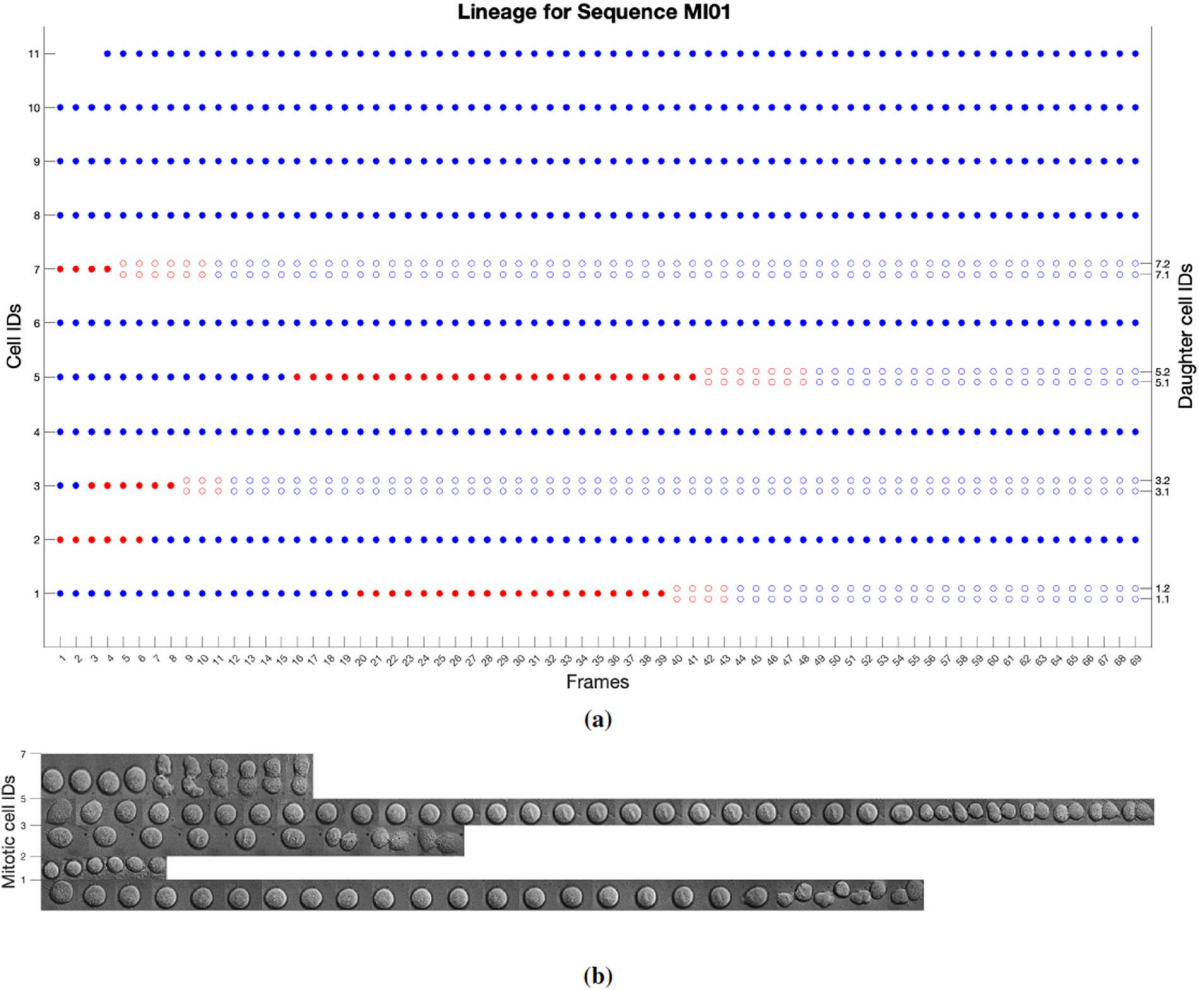
Representation of the lineage information for sequence MI01 obtained using the UseExample 2.m distributed with the dataset (see Usage Notes): (**a**) for each parent cell (cell IDs on the left y-axis), a full dot (blue for interphase cells, red for mitotic cells) represents its appearance in a sequence image (frame numbers on the x-axis); an empty dot represents one of its daughters (colored according to its interphase or mitotic class); e.g., the cell identified with ID 1 is interphase from frame 1 to frame 19, then it enters mitosis at frame 20 and produces two daughters that appear as cell doublets (late mitosis) from frame 40. The mitosis ends at frame 44 when the two daughter cells become interphases; (**b**) image crops of mitotic cells (IDs on the y-axis) in the sequence; each crop corresponds to a full red dot or a couple of empty red dots of a lineage tree in (**a**).

A few criteria have been adopted for *Task 1* annotations:

- Cells close to the image boundary that are only partially visible at the beginning of the sequence (e.g., the partially visible cell in the top right corner of the images of Fig. 5) have not been annotated. This is the criterion generally adopted by biologists when examining microscopy videos.
- Daughter cells resulting from the division of a mitotic cell (e.g., cells 7.1 and 7.2 in the bottom image row of Fig. 5c), whose morphology is very similar to that of early mitotic cells, are separately segmented and classified as mitotic for a few frames after becoming doublet, until their morphology changes to that of interphase cells. The criterion adopted for daughter cells goes toward higher generalizability of the provided annotations, which can be readily used for evaluating cell segmentation and classification in both single images (where time information is not taken into account) and time-lapse sequences. Nonetheless, the correct biological identification of daughter cells is stored via the lineage information (the “Parent” column of the annotation file, as described in the Data Records section), where daughter cells are associated with their parent cell only as soon as they become interphases, as exemplified in Fig. 6. Further lineage analysis along the following generation is feasible in our dataset using the sequence MI06 which covers 23 hours of video recording from pre-synchronized cultures.

Table 2 reports detailed information on the annotations for *Task 1*, including cell line, sequence duration, number of frames, number of annotated frames, number of annotated cells in each of the considered mitotic and interphase classes, total number of annotated cells in all the sequence frames, and total number of tracked cells, also considering the lineage traced ones, for each sequence.

#### *Task 2*: Discriminating non-interphase cells into four phenotype classes

For this task, only non-interphase cells have been taken into account and classified as belonging to four different classes based on the cell morphology observed under DIC live imaging: Early Mitoses, including prometa- and metaphases, identified as cells which rounded-up with condensed chromosomes; Late Mitoses, including ana- and telophases identified as elongated oval cells with two chromosome sets and as rounded doublets; Multipolar division, identified as a cell that divides in more than a doublet; and Cell Death, identified by membrane blebbing and cell shrinkage. A concise description coupled with single-cell image examples of the four classes analyzed in this task is given in the lower part of Fig. 4.

**Fig. 7:**
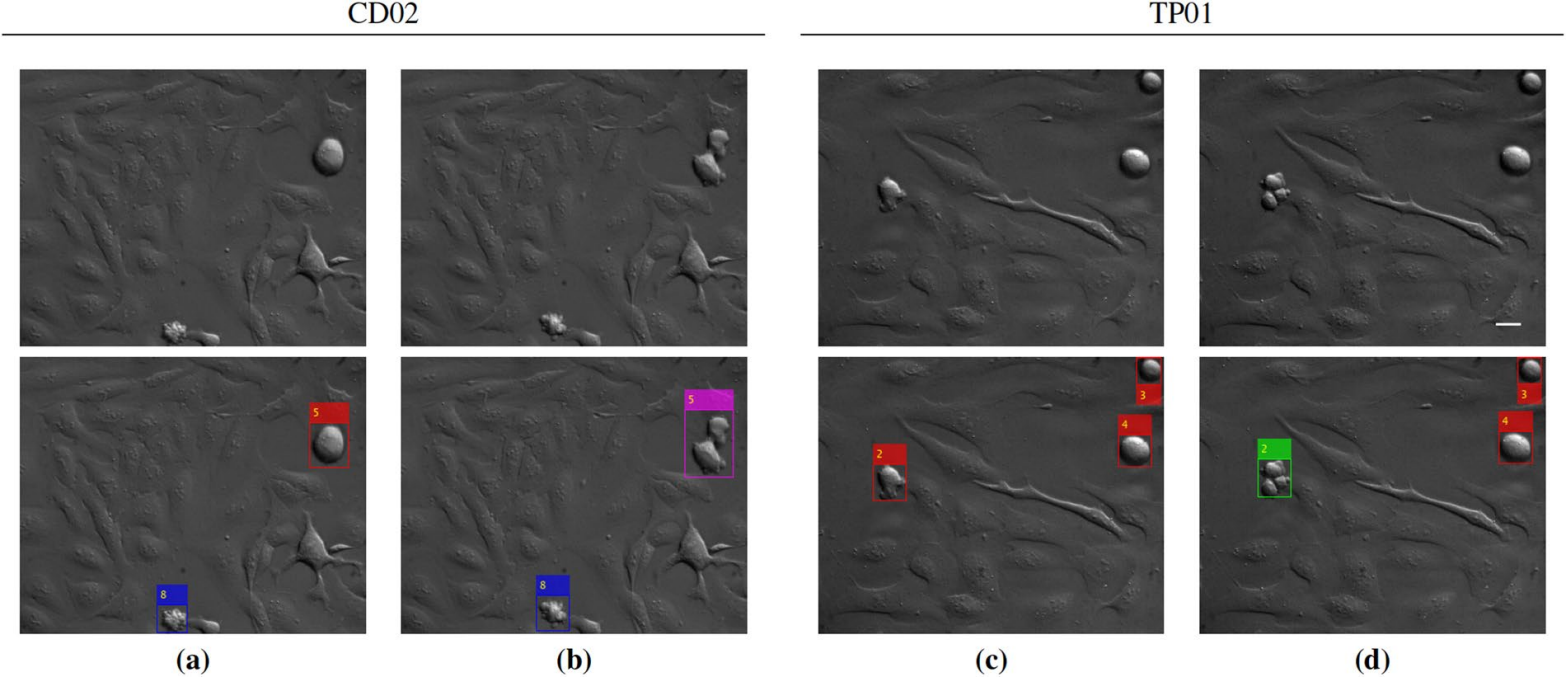
Example of *ALFI* dataset annotations for *Task 2*. Sequences CD02 (frames no. 49, 50 (**a,b**)) and TP01 (frames no. 26, 27 (**c,d**)) and their annotations (second row). Bounding boxes and tracking IDs are used to identify and track cells classified as Early Mitosis (red), Late Mitosis (magenta), Multipolar (green), or Cell Death (blue). Scale bar: 25 *μ*m.

The annotations for this task consist of bounding boxes and tracking information for sequences MI01-MI08 (containing only Early and Late Mitoses), CD01-CD09 (containing also several Cell Death events), and TP01-TP12 (containing also several Multipolar divisions, mainly tripolar). Examples of annotations superimposed over the corresponding images are given in Fig. 7 for the CD02 and TP01 sequences. The bounding box annotations have been produced using the makesense.ai online tool^25^, while tracking has been obtained using the same procedure adopted for *Task 1*.

For *Task 2* too, a few criteria have been adopted for the annotations:

- Interphase cells have not been annotated. Indeed, attention is given only to the four described phenotype classes for this task.
- The four considered phenotype events generally last for a few consecutive frames and may not necessarily be correctly classified by looking only at single frames. However, in light of higher generalizability, annotations are provided for every single frame for the entire duration of the event.
- As for *Task 1*, cells close to the image boundary that are only partially visible at the beginning of the sequence (e.g., the partially visible mitosis close to the bottom image boundary of Fig. 7a,b) have not been annotated.
- Contrary to the annotations for *Task 1*, Late Mitoses (e.g., the cell with ID 5 in Fig. 7b, magenta box) are annotated with a single bounding box, and no lineage information is provided. Therefore, for sequences MI01-MI08 the two types of annotations (for *Tasks 1* and *2*) are slightly different.

Table 3 reports detailed information on the annotations for *Task 2*, including cell line, sequence duration, number of frames, number of annotated frames, number of annotated cells for each of the four considered phenotype events, total number of annotated cells in all the sequence frames, and total number of tracked cells for each sequence.

## Data Records

The *ALFI* dataset includes videos, images, and annotations for 29 label-free microscopy sequences named MI01-MI08, CD01-CD09, and TP01-TP12, obtained as described in the Methods section. The dataset is made publicly available to the scientific community through figshare^19^. The main dataset directory includes the ALFI_dataset_Readme.txt file, containing detailed metadata describing the dataset structure and content, and three directories:

- Data&Annotations, containing images and annotations for all the 29 sequences, as described below;
- Videos, containing all the 29 videos (named CD01.nd2,…, TP12.nd2);
- UseExamples, containing usage examples, as described in the Usage Notes.

**Fig. 8:**
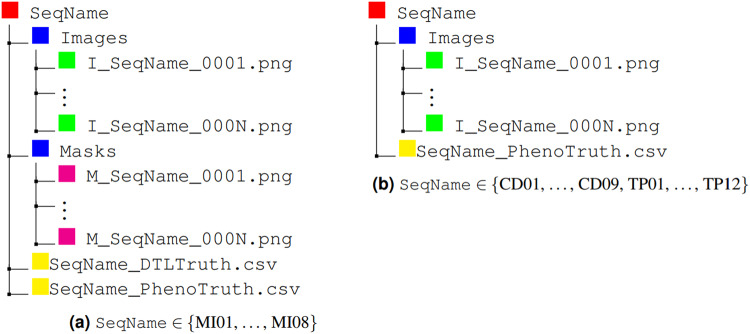
Directory trees for the generic sequence SeqName included in the Data&Annotations directory, diversified for (**a**) sequences MI01-MI08, including annotations for both *Task 1* (Masks and SeqName_DTLTruth.csv) and *Task 2* (SeqName_PhenoTruth.csv); (**b**) sequences CD01-CD09 and TP01-TP12, including only annotations for *Task 2* (SeqName_PhenoTruth.csv).

The content for the generic sequence SeqName in the Data&Annotations directory can be described via the directory trees shown in Fig. 8:

- All the images are included in the Images directory; they are numbered starting from 1 and are given as PNG files, 16-bit depth, and 1280 × 1024 pixel resolution.
- The segmentation masks for *Task 1*, available only for sequences MI01-MI08, are included in the Masks directory; they are numbered according to the sequence image they refer to and are given as PNG files, 8-bit depth, and 1280 × 1024 pixel resolution. Background, interphase cells, and mitotic cells are labeled using values of 0, 128, and 255, respectively. The related bounding box, tracking, and lineage annotations are included in the SeqName_DTLTruth.csv file. Here, for each annotated cell, information is provided on: number of sequence image, cell ID, cell class (either Interphase or Mitosis), bounding box, specified by its upper left corner and its dimensions, and parent cell ID, as summarized in Table 4a. These annotations allow the evaluation of cell segmentation (via the segmentation masks), classification (using the cell class), tracking (using the cell ID), and lineage (using the cell parent ID).

**Table 4:** Annotation tags for *Task 1* and *Task 2*. (**a**) *Task 1* (SeqName_DTLTruth.csv). Example: the line [10, 5.1, Interphase, 3, 3, 10, 15, 5] in the SeqName_DTLTruth.csv file indicates at frame 10 the interphase cell 5.1, with bounding box having top-left corner in (3,3) and size 10 × 15 pixels, whose parent is cell 5. (**b**) *Task 2* (SeqName_PhenoTruth.csv). Example: the line [20, 11, LateMitosis, 1, 9, 10, 18] in the SeqName_PhenoTruth.csv file indicates at frame 20 the late mitosis cell 11, with bounding box having top-left corner in (1,9) and size 10 × 18 pixels.

Tag	Description
(a)	
ImNo	number of sequence image
ID	cell ID
Class	cell class (Interphase or Mitosis)
xmin	x coord. of top-left corner of the bounding box
ymin	y coord. of top-left corner of the bounding box
width	width of the bounding box
height	height of the bounding box
Parent	ID of the parent cell
**(b)**	
ImNo	number of sequence image
ID	cell ID
Class	cell class (EarlyMitosis, LateMitosis, Multipolar, or CellDeath)
xmin	x coord. of top-left corner of the bounding box
ymin	y coord. of top-left corner of the bounding box
width	width of the bounding box
height	height of the bounding box
- Annotations for *Task 2* for all the sequences are included in the SeqName_PhenoTruth.csv file. Here, for each annotated cell, information is provided on: number of sequence image, cell ID, cell class (including EarlyMitosis, LateMitosis, Multipolar, and CellDeath), and bounding box, specified by its upper left corner and its dimensions, as summarized in Table 4b. The parent cell ID is not specified, since, for these sequences, interphase cells are not tracked. These annotations allow the evaluation of cell detection (using the bounding boxes), phenotype classification (using the cell class), and tracking (using the cell ID) only for non-interphase cells.

## Technical Validation

**Fig. 9:**
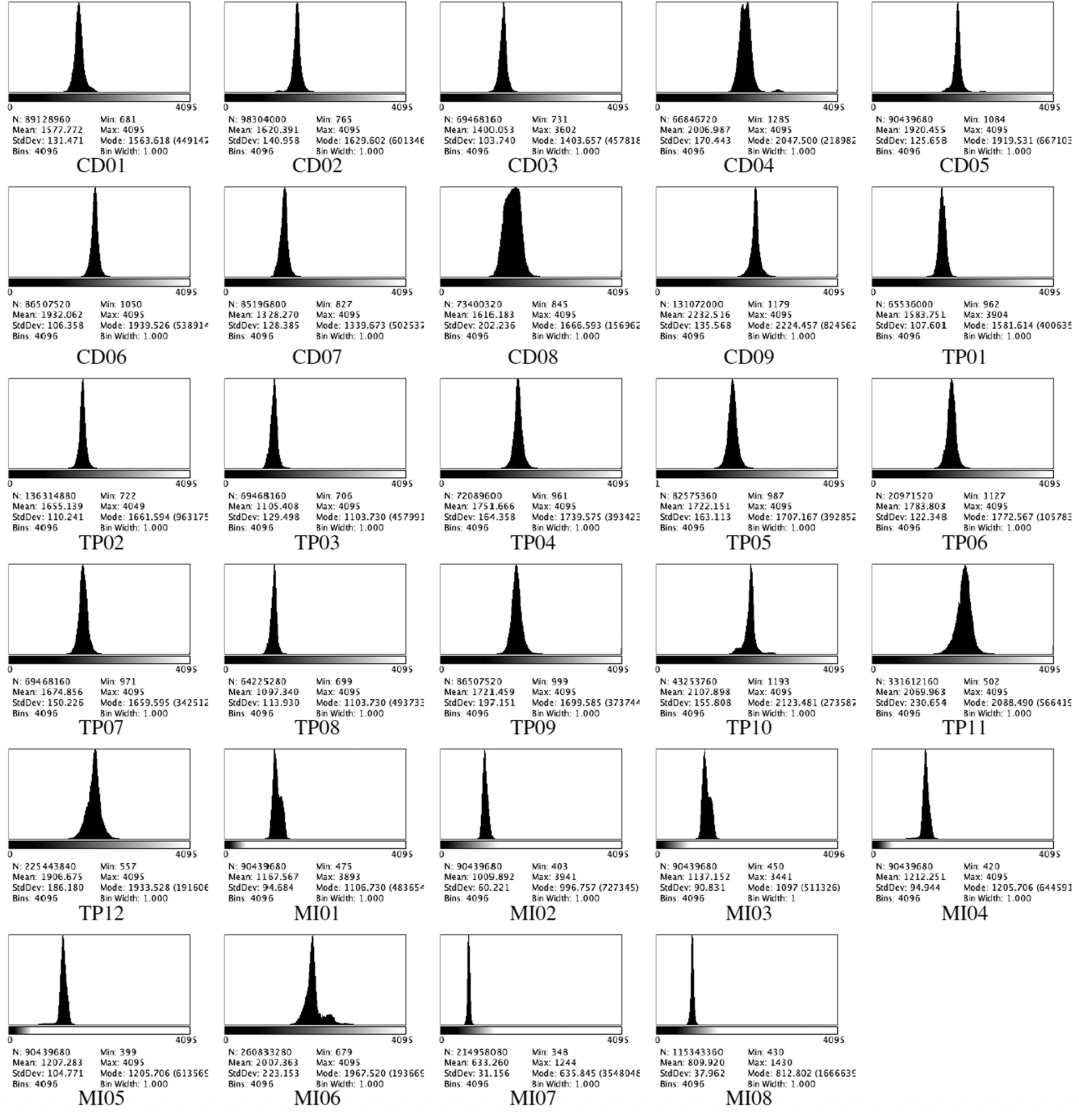
*ALFI* original videos validation. The histograms show the distribution of grey-scale pixel values of original sequences. Since a single frame has a 12-bit depth, the number of bins was set to 4096, and bin width was automatically assigned to 1 for all videos. The graphs also display the total number of pixels, minimum, maximum, mean, mode, and standard deviation values for each pixel distribution.

The overall quality of *ALFI* videos was analyzed through the histograms of pixel intensity values reported in Fig. 9, carried out using the image processing package Fiji^22^. The collected intensity values distributions have a minimum value between 348 and 1285, while the maximum value ranges between 1244 and 4095. Overall mean values range between 633 and 2107. The different standard deviation values point out the different scattering to the pixel values around the minimum values of each sequence.

A board of four biologists with more than ten years of experience reviewed the quality of all the images and annotations of the *ALFI* dataset. For sequences where fluorescence was present, we also carefully checked that our manual segmentation of interphase nuclei precisely overlapped with the GFP signal of H2B-GFP.

## Usage Notes

To assist other researchers with the reuse of our data, in the UseExamples directory, we provide two use examples:

1. UseExample1.m is a Matlab script for plotting the bounding box annotations over the original sequence images, as shown in Fig. 7. This use example is available also in Python (UseExample 1.py), with the related Readme file (UseExample 1.py.readme.txt). The output for sequence TP06 is included in the UseExample1Out directory.
2. UseExample2.m is a Matlab script for plotting a representation of cell lineage for sequences MI01-MI08, together with image crops of the involved cells, as shown in Fig. 6. It is optionally possible (via the OnlyMitoses input parameter) to plot the lineage representation only for mitotic cells; this can be useful for highly populated sequences, such as MI07 and MI08, where the plot of Fig. 6a would appear too dense. It should be observed that the provided lineage representation is suitable only for the first generation of daughter cells, as otherwise the plot (e.g., the one in Fig. 6a) would become too complex for multiple generations. The output images for all sequences MI01-MI08 are provided in the UseExample2Out directory.

## Data Availability

The NIS-Elements Viewer software (v. 4.11.0, www.microscope.healthcare.nikon.com) was used to automatically equalize the videos (AutoScale LUTs tool) and extract individual frames (see *ALFI* dataset), that were converted to the PNG format using the Fiji^22^ (https://fiji.sc) distribution of ImageJ^23^ (https://imagej.nih.gov/ij). The ilastik tool (v. 1.3.3, www.ilastik.org) was used to produce initial semantic segmentations of interphase nuclei and mitotic cells for sequences MI01-MI08 (see *ALFI* annotations for *Task 1*) using the Pixel Classification workflow. The workflow performed the following steps: (1) load the selected input image sequence; (2) define three user-driven classes: *mitosis*, *interphase*, *background*; (3) define pixel filters, based on color, intensity, and texture, as image features at different scales; (4) learn examples for each class interactively by user-defined brushstrokes with classes colors on one or more selected input images; (5) classify image pixels for the whole sequence using output filters, and a Random Forest classifier^26^; (6) correct pixel classification interactively repeating steps 4-5); (7) save the output. Matlab (v. R2020a, www.mathworks.com) was used to (1) refine the initial masks (see *ALFI* annotations for *Task 1*) using the Video Labeler App; (2) extract the bounding boxes from the masks (see *ALFI* annotations for *Task 1*) using the regionprops function; (3) associate the bounding boxes of the same object in adjacent frames based on motion, estimated by a Kalman filter (see *ALFI* annotations for *Task 1* and *Task 2*); and (4) produce the use examples (see Usage Notes). The bounding box annotations have been produced using the makesense.ai online tool^25^ (https://github.com/SkalskiP/make-sense).
